# Effect of Vaccination on *Bordetella pertussis* Strains, China

**DOI:** 10.3201/eid1611.100401

**Published:** 2010-11

**Authors:** Liu Zhang, Yinghua Xu, Jianhong Zhao, Teemu Kallonen, Shenghui Cui, Yunqiang Xu, Qiming Hou, Fengxiang Li, Junzhi Wang, Qiushui He, Shumin Zhang

**Affiliations:** Author affiliations: National Institute for the Control of Pharmaceutical and Biological Products, Beijing, People’s Republic of China (L. Zhang, Y. Xu, S. Cui, Q. Hou, F. Li, J. Wang, S. Zhang);; Hebei Medical University, Shijiazhuang, PRC (J. Zhao);; The Second Hospital of Shijiazhuang City, Shijiazhuang (Y. Xu); National Institute for Health and Welfare, Turku, Finland (T. Kallonen, Q. He);; University of Turku, Turku (T. Kallonen); 1 These authors contributed equally to this article.

**Keywords:** China, Bordetella pertussis, whooping cough, pertussis, incidence, vaccination, genotyping, PFGE, bacteria, research

## Abstract

Whole-cell pertussis vaccine was introduced in China in the early 1960s. We used standard typing methods to compare 96 *Bordetella pertussis* isolates collected before and after introduction of vaccination, during 1953–2005. The following vaccine-type alleles of the pertussis toxin (*ptx*) gene were characteristic for all prevaccination strains: *ptxA2*, *ptxA3,* and *ptxA4.* The shift to *ptxA1* occurred since 1963. All isolates collected since 1983 contained *ptxA1*. Pertactin (*prn*) allele 1, *prn1,* was predominant, although *prn2* and *prn3* have been detected since 2000. Serotypes fimbriae (Fim) 2 and Fim2,3 were found in all isolates collected before 1986. During 1997–2005, Fim3 became prevalent. Although changes in electrophoresis profiles over time were observed, the predominant profiles during 1997–2005 resembled those during the prevaccine era and those found in Europe before the 1990s. *B. pertussis* strains in China may differ from those in countries that have a long history of high vaccine coverage.

## Abstract 2

Strains in China may differ from those in countries that have long histories of high vaccination coverage.

Whooping cough (pertussis) is an acute respiratory infectious disease caused by the bacterium *Bordetella pertussis*. After the whole-cell pertussis (Pw) vaccines were introduced in many countries during 1940–1960, illness and death rates from pertussis have decreased dramatically (*1**,**2*). However, pertussis remains a leading cause of vaccine-preventable deaths worldwide (*1*). A resurgence of pertussis has been observed in developed countries despite high vaccination coverage (*3**–**9*).

In the People’s Republic of China, vaccination against pertussis was started in the early 1960s, when 3 doses of Pw vaccine combined with diphtheria and tetanus toxoids were given at 3, 4, and 5 months of age. In 1982, a booster dose at 18 months of age was added (*10**,**11*). Pw vaccines are free of charge in China. Since 1995, acellular pertussis (Pa) vaccines containing purified pertussis toxin (Ptx) and filamentous hemagglutinin have been also introduced. However, because Pa vaccines are offered at the patient’s expense, use of these vaccines has been minimal, especially in some resource-limited areas. Although since 2007 Pa vaccines have been included in the national expanded program on immunization, Pa and Pw vaccines are still used in most provinces because of limited availability and cost of Pa vaccines. Although the reported vaccination coverage for the primary 3 doses increased with time, before the 1980s it was low. From 1983 through 2008, coverage ranged from 58% to 99% (median 93%) (Figure 1) (*12*).

**Figure 1:**
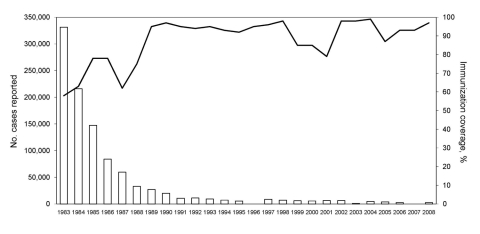
Number of reported pertussis cases and pertussis vaccination coverage in China, 1983–2008 (*12*). Although vaccination coverage increased with time, it was low before the 1980s and only 58% in 1983.

In China, pertussis is a reportable infectious disease, and the number of reported cases has been decreasing (Figure 1). Since the 1990s, incidence has been <1 case/100,000 population (*12*). In China, pertussis is clinically diagnosed by physicians; laboratory methods such as culture, PCR, and serologic analysis are not used for diagnosis of pertussis. Therefore, the reported low incidence may be related to the diagnostic criteria used, suggesting substantial underreporting. Pertussis remains endemic to China (*10**–**12*), and a local outbreak was reported in April 1997.

In many countries, divergence in major antigens Ptx, pertactin (Prn), fimbriae (Fim) 2, Fim3, and tracheal colonization factor (TcfA) between the vaccine strains and circulating isolates has been reported (*3**,**4**,**7**,**9**,**13**–**16*). Furthermore, marked changes in *B. pertussis* strains have been found in these countries after introduction of vaccination. To learn more about the *B. pertussis* strains circulating in China, we used standardized typing methods to analyze and compare *B. pertussis* isolates collected before and after the introduction of vaccination (*17*).

## Materials and Methods

### Bacterial Strains and Culture

**Figure A1:**
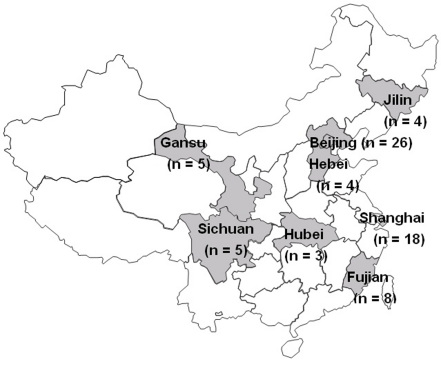
Distribution of the *Bordetella pertussis* isolates collected during 1953–2005 in China. Places where the isolates were recovered are indicated by shading, and the numbers of isolates obtained from each location are provided within parenthesis. Of the 96 isolates studied, no information about origin was available for no. 23.

We tested 3 vaccine strains and 96 clinical isolates: 25 isolates from 1953–1958, 52 from 1963–1985, and 19 from 1997–2005. These 3 periods were chosen according to when Pw vaccines were introduced (1960) and when Pa vaccines were added to the vaccination program (1995). Information on vaccine strains and clinical isolates is shown in Figure A1 and in the Technical Appendix. Clinical information for patients from whom *B. pertussis* was isolated was not available. Vaccine strains P3s10 and CS were isolated in Beijing in 1951 (Figure A1). Vaccine strain 18530 originated in the United States. Strains P3s10 and 18530 have been used to produce Pw vaccines since the early 1960s, and strain CS has been used to produce Pa vaccines since 1995. All *B. pertussis* isolates were confirmed by slide agglutination with specific antiserum to *B. pertussis* and *B. parapertussis* (Murex Diagnostics, Dartford, UK) and by PCR according to insertion sequence *IS481* (*18*). *B. pertussis* strains were grown on Bordet-Gengou agar supplemented with 15% defibrinated sheep blood, incubated at 37°C for 4–5 days.

### Serotyping

Serotyping was performed by a microtiter plate–based monoclonal agglutination assay as described (*17*). Monoclonal antibodies against Fim2 (NIBSC 04/154) or Fim3 (NIBSC 04/156) were used.

### DNA Sequencing

PCR-based sequencing of 5 genes (*prn, ptxA, tcfA, fim2*, and *fim3*) was performed as described (*17**,**19**,**20*) with minor modifications. Nucleotide sequences were determined for the complete opening read frames of *ptxA*, *fim2*, *fim3*, and *tcfA,* and for part of *prn*. The same primers were used for amplification and sequencing (Table). The sequencing assays were performed with an ABI Prism 3100 DNA sequencing system (Applied Biosystems, Foster City, CA, USA), and data analysis was conducted with DNASTAR (Madison, WI, USA) software.

**Table:** Oligonucleotide primers designed and used for PCR amplification and sequencing of *Bordetella pertussis*, China*

Primer	Sequence, 5′ → 3′	Gene	Position
prn-1F	ATGTCTCTGTCACGCATTGTCA	*prn*	151–172
prn-1R	GTCCTGCATGACGACCAGCTTG	*prn*	1653–1632
prn-2F	CTCGAACGTCGGTGCGCTAC	*prn*	1479–1498
prn-2R	TCGCGTCCAGGTAGAAACCG	*prn*	2347–2328
ptxA-F	GGCACCATCAAAACGCAGAGGGG	*ptxA*	476–498
ptxA-R	ATTACCGGAGTTGGGCGGGGCTG	*ptxA*	1347–1325
fim2-F	ACCCATGCAAATCCCTTTCCAACGC	*fim2*	177–201
fim2-R	GGGGGTTGGCGATTTCCAGTTTCTC	*fim2*	877–853
fim3-F	ATGTCCAAGTTTTCATACCCTGCCT	*fim3*	336–360
fim3-R	TTCGTCTCCTGACGCCGCGTAGCGG	*fim3*	1033–1009
tcfA-1F	CCACATTGATTCAGGCCGCT	*tcfA*	251–270
tcfA-1R	CGTCCGCAGGAGACTTGGAA	*tcfA*	1096–1077
tcfA-2F	GACTCCGGTATGTCCGATTC	*tcfA*	976–995
tcfA-2R	CTACCAGGCGTAGCGATAGC	*tcfA*	2346–2327

*Primer positions are listed according to the numbering of the sequences of the following GenBank accession nos.: *ptxA*, M13223; *prn*, J04560; *fim2*, Y00527; *fim3*, X51543; and *tcfA*, U16754. prn, pertactin; ptx, pertussis toxin; fim, fimbriae; tcf, tracheal colonization factor.

### Pulsed-Field Gel Electrophoresis

Pulsed-field gel electrophoresis (PFGE) was performed as recommended (*17*) with minor modifications. Briefly, after the DNA plugs were treated with 50 U of *Xba*I (New England Biolabs, Ipswich, MA, USA), the cleaved DNA fragments were separated by electrophoresis on a 1% agarose gel by using a Chef Mapper (Bio-Rad, Hercules, CA, USA) with pulse times of 2.16–44.69 s for 24 h. The band patterns were analyzed with BioNumerics program version 4.0 (Applied-Maths, Kortrijk, Belgium). The clustering method used was the unweighted pair group method with arithmetic mean. The Pertussis Reference Laboratory of the National Institute for Health and Welfare, Turku, Finland, used PFGE to retest 53 clinical isolates, 3 vaccine strains, and international reference strain 18323 (*7*).

The selection criteria for the 53 isolates included at least 1 strain for 1 unique profile. If 2 strains had identical profiles, both were retested. If multiple strains represented the same profile, the strains isolated in different years or different regions were included. For PFGE, 8 international reference strains were included. The nomenclature was based on the profiles already defined for Finland (BpFINR) and Sweden (BpSR) (*7**,**21*). Profiles assigned as BpCHR have been found only among the analyzed isolates from China. A 2-sided Fisher exact test was used to compare frequencies of strain serotypes and genotypes from the 3 periods.

## Results

### Fimbrial Serotypes

**Figure 2:**
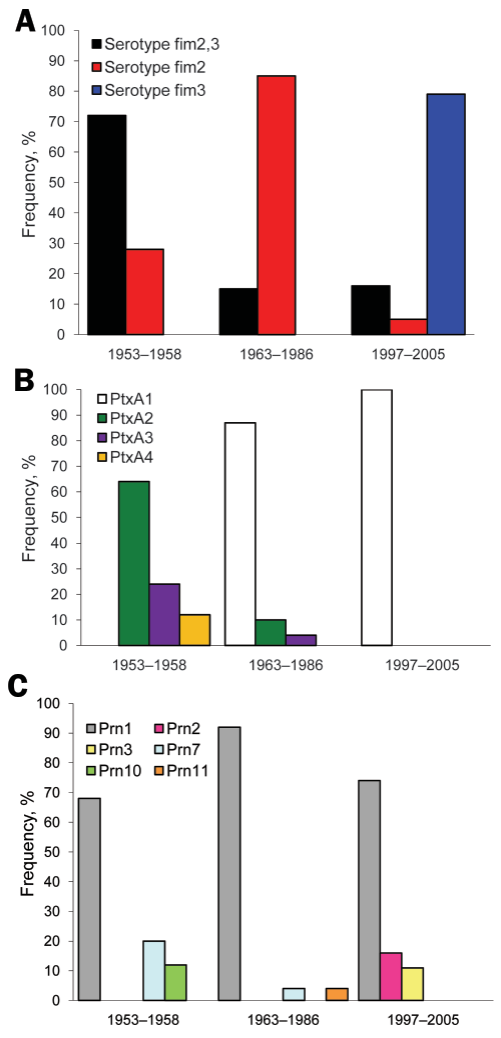
Frequencies of A) fimbrial (*fim*) serotypes, B) pertussis toxin (*ptx*) A alleles, and C) pertactin (*prn)* alleles in *Bordetella pertussis* isolates collected in China during 1953–1958, 1963–1985, and 1997–2005.

Vaccine strains P3s10 and CS were serotype Fim2,3, and vaccine strain 18530 was serotype Fim3. Among the clinical isolates, all 3 serotypes were found (Figure 2, panel A). Significantly more isolates collected during 1963–1986 were serotype Fim2 than were isolates collected during 1953–1958 (p<0.001). Of the 19 isolates collected during 1997–2005, we found that 15 (79%) were Fim3, 3 (16%) were Fim2,3, and 1 (5%) was Fim2. Significantly more isolates collected during 1997–2005 were serotype Fim3 than were isolates collected during 1953–1958 and 1963–1986 (p<0.001 for each).

### Alleles of *ptxA, prn, tcfA, fim2*, and *fim3*

Vaccine strains P3s10 and CS contained *ptxA2*/*prn1*/*tcfA2*/*fim2–1*/*fim3–1*, whereas vaccine strain 18530 contained *ptxA3*/*prn1*/*tcfA2*/*fim2–2*/*fim3–1*. Among the clinical isolates, all 4 *ptxA* alleles (*ptxA1*, *ptxA2*, *ptxA3*, and *ptxA4*) were found (Figure 2, panel B). However, the frequency of each allele changed over time. In the prevaccine era, 64% (n = 16), 24% (n = 6), and 12% (n = 3) of isolates contained *ptxA2*, *ptxA3*, and *ptxA4*, respectively. The allele *ptxA1* appeared in 1963 and has become predominant since then. After the 1980s, all isolates contained *ptxA1.*

**Figure A2:**
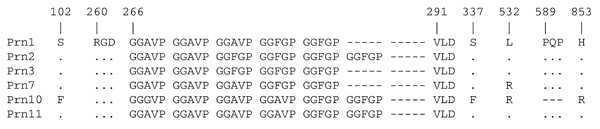
Amino acid sequence of pertactin (Prn) of *Bordetella pertussis* from China. Numbering is relative to the first methionine of Prn1. Dots represent identical sequences and dashes represent deletions. The RGD (arg-gly-asp) motif implicated in adherence to host receptors is indicated.

Altogether 6 *prn* alleles (*prn1, prn2, prn3, prn7, prn10*, and *prn11*) were detected. The amino acid sequences in variable regions of the 6 alleles are shown in Figure A2. Allele *prn1* remained predominant at 81% (78/96) during the study period (Figure 2, panel C). No significant difference was found in the frequency of finding *prn2* and *prn3* in isolates collected during 1997–2005 than in those collected during 1953–1958 (p = 0.749) and 1963–1986 (p = 0.0513). All 7 isolates with *prn7* contained *ptxA3*, whereas all 3 isolates with *prn10* contained *ptxA4*. Two isolates with *prn11* contained *ptxA2*. All 5 isolates with *prn2* or *prn3* contained *ptxA1*.

Four *tcfA* alleles (*tcfA1, tcfA2, tcfA5*, and *tcfA9*) were identified. Allele *tcfA2* was most common, representing 94% (n = 90) of the isolates (Technical Appendix). Also detected were 2 *fim2* (*fim2–1* and *fim2–2*) and 3 *fim3* (*fim3–1*, *fim3–2*, and *fim3–4*) alleles. Of the 96 isolates, 90 (94%) contained *fim2–1*. All 6 isolates with *fim2–2* were recovered during 1953–1958. Five of the 6 isolates with *fim2–2* contained *prn7*. For the *fim3* alleles, 96% of isolates were *fim3–1*. All 3 isolates with *fim3–2* and 1 isolate with *fim3–4* were recovered in 1997–2005. Identical amino acid sequences were found for *fim3–1* and *fim3–4*, but a silent mutation at nt 87 was found for *fim3–4*. All 3 isolates harboring *fim3–2* contained *prn2*.

### PFGE Profiles

**Figure 3:**
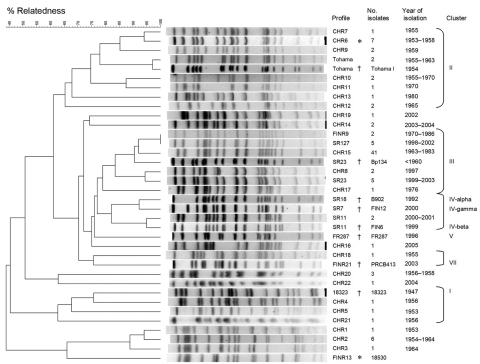
Dendrogram analysis of 27 pulsed-field gel electrophoresis profiles of *Bordetella pertussis* isolates circulating in China during 1953–2005. The unweighted pair group method with arithmetic mean with 1% band tolerance and 1% optimization settings was used as the clustering method. * indicates international reference strains (*17**,**23*); † indicates vaccine strains from China. Vaccine strains P3s10 and CS represent BpCHR6, and vaccine strain 18530 represents BpFINR13.

Vaccine strains P3s10 and CS had an identical profile, BpCHR6, whereas vaccine strain 18530 represented BpFINR13 (Figure 3). The 96 isolates produced 27 distinct profiles, 4 of which (BpFINR9, BpSR127, BpSR23, and BpSR11) have been reported in Europe. The PFGE profiles obtained from vaccine strain 18530 (BpFINR13) and international reference strain 18323 were not detected among the isolates from China. The 6 common profiles represented 70% of isolates (41 isolates with BpCHR15, 7 with BpCHR6, 6 with BpCHR2, 5 with BpSR127, 5 with BpSR23, and 3 with BpCHR20). The PFGE profiles changed over time. Dendrogram analysis of the 27 profiles indicated that they belonged to 8 clusters. Among these clusters, 5 (clusters I–IV and VII) have been reported in Europe (*5**,**22**,**23*).

Previous studies have shown that cluster I includes international reference strain 18323 and 1 clinical isolate (*21**,**22*). Clusters II and III include the vaccine strains and most strains that were circulating before the 1990s. Cluster IV contains most strains currently circulating in Europe. Cluster VII, a new group, consists of some isolates collected from Finland in 2004 (*23*). Of the 3 clusters identified in our study, 1 consisted of 4 profiles (BpCHR1–3 and BpFIN13). Profile BpFINR13 was found only in pertussis vaccine strain 18530 (*7*). The strain was obtained from the United States and used as a vaccine strain in Finland and China. The second cluster contained profiles BpCHR14 and BpCHR19, and the third contained profiles BpCHR20 and BpCHR22.

In this study, 56 isolates tested belonged to cluster III (Figure 3). Cluster III contained 6 profiles (BpCHR8, BpCHR15, BpCHR17, BpFINR9, BpSR23, and BpSR127) with a minimum of 79% overall relatedness. All isolates belonging to the cluster were collected during 1963–1986 and 1997–2005. Of the 96 clinical isolates, only 2 isolates that belonged to cluster IV (IV-β) were identified; these 2 isolates had PFGE profile BpSR11. Strains with BpSR11 were first detected in France in 1996 (*22*) and have been prevalent in Europe since then (*13*). The 2 isolates with BpSR11 were recovered in 2000 and 2001 and contained *prn2*.

## Discussion

Few *B. pertussis* isolates from China contained nonvaccine type alleles *prn2* or *prn3*; those that did were found later. In many countries, the *prn1* allele is found in most vaccine strains and predominated before introduction of vaccination. However, the vaccine type strains were gradually replaced by nonvaccine type strains, mainly with allele *prn2*, after the introduction of vaccination. The most prevalent type in modern isolates is *prn2* (*7**,**14**,**20**,**24*). In Taiwan, Pw vaccines have been offered since 1954 (*25*). When 168 clinical isolates collected in Taiwan during 1993–2004 were analyzed, *prn2* strains accounted for 39% in 1993–1996 and increased to 90% in 1998–2004. In contrast to findings for many countries with long histories of high vaccination coverage, *prn2* was first found in China in 2000. The exact reasons for the low frequency of strains with *prn2* and their relatively late emergence in China are not known. One explanation might be the low vaccination coverage in China before the 1980s and differences in vaccine coverage between urban and rural areas. Another reason might be the closed borders.

In Japan, divergence of Prn and Ptx between vaccine strains and circulating isolates (*26**–**28*) has been reported. Pw vaccines were introduced in Japan in 1958; the vaccine strain used was *prn1*/*ptxA2*. In 1971, reported vaccination coverage was 50% (*8*). In 1976, vaccination coverage dropped to only 9%, and pertussis returned. In 1981, a Pa vaccine containing Ptx and filamentous hemagglutinin was introduced in Japan (*27*). The strain used for production of the Pa vaccines was Tohama I (*prn1*/*ptxA2*), isolated in the 1950s. When 107 isolates collected from 1988 through 2001 were studied, the nonvaccine type *prn2*/*ptxA1* appeared in 1994 and was found in 27%–42% of isolates from 1994 through 2001 (*26*). A recent study reported similar frequency (33%) of the nonvaccine type *prn2/ptxA1* in Japan when 60 isolates collected during 1991–2007 were analyzed (*28*).

TcfA has been shown to be crucial for *B. pertussis* colonization (*29*). A total of 9 *tcfA* alleles have been reported (*30**,**31*), and the most common allele is *tcfA2* (*20**,**24*). Our finding that 94% of isolates studied contained *tcfA2* agreed with findings from earlier studies (*20**,**24*). Allele *tcfA1* has been described only for reference strain 18323. In our study, 3 clinical isolates recovered during 1953–1958 were found to contain *tcfA1*. All 3 isolates were recovered from the same geographic area. Allele *tcfA1* contains a 75-bp segment not found in other *tcfA* variants. The strain with *tcfA1* was postulated to be the progenitor of the strains with *tcfA2*, *tcfA3,* or *tcfA5* (*20*).

Several studies have demonstrated that serotype Fim3 is predominant in vaccinated populations, whereas serotypes Fim2 or Fim2,3 are predominant in unvaccinated populations (*14**,**32**,**33*). In Sweden, before 1979 when Pw vaccines were first used, 70% of circulating strains were serotype Fim3 (*32*). During 1979–1995, when pertussis vaccination was stopped, Fim2 started to increase and reached 64% in the early 1990s. In 1996, when general vaccination with Pa vaccines was reintroduced, prevalence of Fim2 declined and Fim3 strains emerged rapidly. In 2002 and 2003, Fim3 was found in 96% of fully vaccinated persons. In China, before introduction of vaccination, the prevalent serotypes were Fim2,3 and Fim2. After vaccination, the frequency of serotype Fim2,3 decreased and Fim2 became predominant. The possible explanation for the predominance of Fim2 strains after vaccination is that the 2 vaccine strains used in China express Fim2,3 and Fim3. The shift from serotype Fim2 to Fim3 was observed in 1998 and coincided with the introduction of Pa vaccines in this country. Pa vaccines without fimbrial antigens may have some effect on fimbrial serotypes of circulating isolates, as was observed in Sweden (*32*); however, the exact reason remains to be shown.

In our study, most strains from China had different PFGE profiles than did those from Europe. However, many PFGE profiles detected among the strains from China fell into the same clusters as those reported in Europe (*5**,**22*). For example, the most common profile, BpCHR15, fell into the same cluster (cluster III) as BpSR23 and BpSR127 (*14*). Cluster III includes most strains circulating in Europe before the 1990s (*5**,**32*). In China, strains with BpCHR15 had been prevalent during 1965–1983. Although the strains with BpCHR15 were recovered from several different regions and over 20 years, the possibility that some strains were isolated during outbreaks cannot be excluded.

The major PFGE cluster circulating in Europe since the 1990s is cluster IV (*5**,**32*). Cluster IV can be divided into 3 subgroups (α, β, and γ), the frequency of which differs among countries. However, since the late 1990s in many countries in Europe, subgroup IV-β became more prevalent than the other 2 subgroups (*13*). In our study, only 2 isolates with BpSR11 belonged to group IV-β, whereas no isolates fell into subgroups IV-α or IV-γ. This PFGE result correlates with genotyping results.

When we examined the association of 6 common PFGE profiles with different allele combinations, we found that of the 51 isolates with BpCHR15, BpSR23, or BpSR127, 94% contained *ptxA1*/*prn1*/*tcfA2*/*fim2–1*/*fim3–1*. Of the 10 isolates with BpCHR6 or BpCHR20, 100% contained *ptxA2*/*prn1*/*tcfA2*/*fim2–1*/*fim3–1.* Of the 6 isolates with BpCHR2, all contained *ptxA3*/*prn7*/*tcfA2*/*fim2–2*/*fim3–1*.

The emergence of *B. pertussis* strains carrying a novel allele (*ptxP3*) for the Ptx promoter has been recently observed in countries with long histories of high vaccination coverage, such as the Netherlands (*34*). Furthermore, all strains from the Netherlands with BpSR11 were found to carry the *ptxP3* allele. In our study, only 2 isolates from China were found to have BpSR11, suggesting that *ptxP3* strains are not common in China.

The limitations of this study include the small number of *B. pertussis* isolates collected during the study period and recent isolates collected mainly from Beijing and its surrounding area. Therefore, our data should be interpreted with caution, and more epidemiologic studies with a larger number of isolates should be conducted in this country.

In conclusion, *B. pertussis* strains in China may differ from those in countries with long histories of high vaccination coverage. Continuous monitoring of *B. pertussis* strains is needed.

## Supplementary Material

Technical Appendix Characteristics of the Bordetella pertussis vaccine strains from China and B. pertussis isolates analyzed..
